# γ-Oryzanol-Loaded PLGA nanoparticles: enhanced drug delivery and therapeutic efficacy for breast cancer therapy

**DOI:** 10.3389/fbioe.2025.1675338

**Published:** 2025-09-03

**Authors:** Teng Ma, Xiaoning Geng, Weiwei Shi, Chunli Yu, Xuesong Wu, Nannan Cui, Ze Zhao, Huazhong Li, Chuanliang Zhao, Qingbin Ni, Xiaodan Zhu, Pengcheng Xia

**Affiliations:** ^1^ Department of Breast Surgery, Taian Central Hospital, Taian, Shandong, China; ^2^ Department of Pharmacy, Taian Central Hospital, Taian, Shandong, China; ^3^ Clinical Laboratory Center, Taian Central Hospital, Taian, Shandong, China; ^4^ Department of General Gynecology, Taian Central Hospital, Taian, Shandong, China; ^5^ Shandong Pharmaceutical Technician College, Taian, Shandong, China; ^6^ Department of Traditional Chinese Medicine Orthopedics, Taian Central Hospital, Taian, Shandong, China; ^7^ Department of Obstetrics and Gynecology, Shandong Provincial Maternal and Child Health Hospital, Jinan, Shandong, China

**Keywords:** γ-oryzanol, PLGA nanoparticles, breast cancer, drug delivery, antitumor effects, molecular mechanisms

## Abstract

**Introduction:**

Breast cancer treatment is plagued by systemic toxicity and drug resistance, prompting the search for better drug delivery systems, with oryzanol, a natural compound with anti-tumor potential but poor water solubility, emerging as a candidate. PLGA nanoparticles, a biodegradable and FDA-approved platform, are designed to encapsulate oryzanol, addressing its solubility issues and enabling targeted, controlled release to enhance anti-breast cancer efficacy. This study focuses on developing and characterizing γ-oryzanol-loaded PLGA (γ-oryzanol@PLGA) nanoparticles, evaluating their formulation, cellular effects, and mechanisms, intending to lay a preclinical foundation for oryzanol as a safe adjuvant therapy for breast cancer.

**Methods:**

To address this unmet need, this study developed γ-oryzanol@PLGA nanoparticles (NPs) as a potential therapeutic strategy. Transmission electron microscopy (TEM) was used to characterize the morphology of the NPs. The colloidal stability and uniformity of nanoparticles were evaluated by measuring the polydispersity index (PDI) and zeta potential. Encapsulation efficiency and loading capacity were determined through UV-visible spectrophotometry. Flow cytometry was employed to assess the cellular uptake of the NPs compared to the free drug, and cytotoxicity assays were conducted to measure the effective concentration. Transcriptomic analysis was performed to identify differentially expressed genes and enriched cancer-related pathways.

**Results:**

TEM results showed that the NPs were spherical with uniform morphology, with blank NPs having a size of 232.50 ± 1.27 nm and drug-loaded NPs being 241.60 ± 7.89 nm. The NPs exhibited excellent colloidal stability (PDI <0.03, zeta potential: −20 to −26 mV). Effective package load (86.22% ± 3.43%) and loading capacity (7.89% ± 0.31%) were achieved. Flow cytometry indicated a 3.2-fold enhanced cellular uptake compared to the free drug at 4 H (*p* < 0.05), and cytotoxicity assays showed a 3-fold reduction in the effective concentration. Transcriptomic analysis identified 576 differentially expressed genes and enriched cancer-related pathways, revealing the molecular mechanisms of the enhanced antitumor effects.

**Conclusion:**

Collectively, these findings demonstrate that γ-oryzanol@PLGA NPs significantly improve drug delivery efficiency and therapeutic potency while maintaining excellent biocompatibility. This presents a promising nanoplatform for breast cancer treatment, warranting further preclinical development. Future studies should focus on *in vivo* validation and the exploration of combination therapies to fully realize the potential of this novel approach.

## 1 Introduction

Breast cancer remains one of the most prevalent malignancies worldwide, accounting for nearly 25% of all cancer cases among women. The global burden of this disease is substantial, with over two million new cases diagnosed annually and approximately 685,000 deaths reported in 2020 alone (Qianru et al., 2024). Current treatment modalities, including chemotherapy, radiation, and targeted therapies, are often limited by systemic toxicity, drug resistance, and high treatment costs that impose significant economic burdens on healthcare systems (Xin et al., 2025). Particularly in triple-negative breast cancer (TNBC), the lack of specific molecular targets results in a poorer prognosis and fewer therapeutic options compared to other subtypes (Giampaolo et al., 2021). These clinical challenges underscore the urgent need for developing novel therapeutic strategies that can improve treatment efficacy while minimizing adverse effects (Shubham D et al., 2025).

γ-Oryzanol, a natural phytochemical derived from rice bran oil, has recently emerged as a promising anticancer agent due to its multifaceted biological activities (Ahmed A et al., 2020). Previous studies have demonstrated its potent antioxidant, anti-inflammatory, and antiproliferative properties against various cancer cell lines (Lin et al., 2019). 24-Methylenecycloartanyl ferulate, a major compound of γ-oryzanol, promotes parvin-beta expression through an interaction with peroxisome proliferator-activated receptor-gamma two in human breast cancer cells (Heon Woong et al., 2015). However, the clinical translation of γ-oryzanol has been hindered by its poor aqueous solubility, low bioavailability, and rapid systemic clearance (Elham et al., 2020). These pharmacokinetic limitations significantly reduce its therapeutic potential despite promising *in vitro* activity, creating a critical gap in natural product-based cancer therapeutics (Kai-Min and Po-Yuan, 2019).

To address these challenges, our study employs poly (lactic-co-glycolic acid) (PLGA) nanoparticles as a drug delivery platform, leveraging their well-established biocompatibility and controlled release properties (Fabienne et al., 2012). PLGA-based nanocarriers offer distinct advantages including enhanced drug solubility, protection from premature degradation, and passive tumor targeting through the enhanced permeability and retention (EPR) effect (Hossein et al., 2025). Recent advances in nanoparticle engineering have further improved drug loading capacity and cellular uptake efficiency, making PLGA an ideal candidate for delivering hydrophobic compounds like γ-oryzanol (Y R et al., 2021). While several studies have explored PLGA nanoparticles for cancer therapy (Tenzin et al., 2023; Lina, 2025), few have systematically investigated their application for natural product delivery combined with comprehensive molecular profiling to elucidate mechanisms of action.

This study utilizes an integrated experimental approach combining advanced material characterization techniques with cutting-edge transcriptomic analysis. We employ transmission electron microscopy (TEM) and dynamic light scattering (DLS) for rigorous nanoparticle characterization, coupled with flow cytometry to quantify cellular uptake dynamics. The transcriptomic profiling using RNA sequencing provides resolution in understanding the molecular pathways modulated by nγ-Oryzanol-Loaded PLGA Nanoparticles. This multimodal methodology offers significant advantages over conventional approaches by simultaneously evaluating physicochemical properties, biological efficacy, and mechanistic insights at the systems level (Michael J et al., 2020).

The primary objectives of this investigation are threefold: (1) To develop and characterize γ-oryzanol-loaded PLGA nanoparticles with optimal physicochemical properties for cancer therapy; (2) To evaluate the enhanced therapeutic efficacy of γ-oryzanol@PLGA Nanoparticles compared to free drug in breast cancer models; (3) To elucidate the molecular mechanisms underlying the observed anticancer effects through transcriptomic analysis. By addressing these objectives, our study aims to establish a proof-of-concept for using PLGA-based nanocarriers to overcome the bioavailability limitations of γ-oryzanol while providing mechanistic insights that could guide future clinical development of natural product-based nanotherapeutics for breast cancer treatment.

## 2 Materials and methods

### 2.1 Preparation of PLGA NPs

PLGA NPs were prepared via the emulsion-solvent evaporation method. Briefly, 100 mg of poly (lactic-co-glycolic acid) (PLGA, MW 12 kDa, LA/GA = 75/25, ester-terminated, purchased from Beijing Thompson Biotechnology Co., Ltd., Beijing, China) was dissolved in 5 mL dichloromethane (DCM) by sonication using an Ultrasonic constant temperature water bath SCQ-H600A (Shengyan Co., Ltd.,Shanghai, China). The PLGA/DCM solution was slowly injected into 25 mL of 2% (w/v) polyvinyl alcohol (PVA, MW ∼67,000, cat. no. P816866-250g, Macklin Biochemical Co., Ltd., Shanghai, China) aqueous solution under vortex mixing. As a non-ionic stabilizer, 2% PVA can inhibit particle agglomeration through steric hindrance effect, while too high concentration will lead to too much PVA residue (affecting biocompatibility), and too low concentration will not stabilize the emulsion. The mixture was sonicated using the sonicator (amplitude 40%, 5 min, 3 s on/3 s off) to form a stable emulsion. This parameter can avoid the excessive fracture of PLGA chain by controlling the droplet breaking energy, while ensuring the formation of a stable emulsion of 100–300 nm (in line with the optimal particle size range of EPR effect). DCM was removed by rotary evaporation under reduced pressure to obtain a nanoparticle suspension, The suspension was centrifuged at 10,000 rpm for 10 min to pellet large, unprocessed PLGA particles (e.g., micrometer-sized aggregates). The supernatant containing fine NPs was carefully transferred to a new tube. The supernatant was diluted with ultrapure water and centrifuged at 15,000 rpm for 20 min to pellet the NPs. The supernatant (containing free PVA) was discarded, and the NP pellet was resuspended in ultrapure water. This washing-centrifugation cycle was repeated 2–3 times to ensure complete removal of residual PVA, which could otherwise interfere with cellular uptake assays.​ For the preparation of unloaded PLGA NPs (control), the same protocol was followed without adding oryzanol during the initial emulsification step. The final NP pellets were resuspended in phosphate-buffered saline (PBS, pH 7.4) for subsequent characterization and *in vitro* studies. This two-step purification strategy ensured the removal of both physical aggregates and soluble stabilizers, yielding homogeneous, PVA-free NPs for accurate evaluation of cellular interactions. For drug-loaded NPs: 100 mg PLGA was mixed with 10 mg γ-oryzanol (>99%, cat. no. O832521-5g, Macklin Biochemical Co., Ltd., Shanghai, China) or 10 mg coumarin 6 (C6, >98%, cat. no. C804226-1g, Macklin Biochemical Co., Ltd., Shanghai, China) in 5 mL DCM, and the above procedure was repeated to prepare γ-oryzanol@PLGA NP and C6@PLGA NP, respectively.

### 2.2 Characterization of NPs

Size, polydispersity index (PDI), and zeta potential of PLGA NP and γ-oryzanol@PLGA NP were determined by dispersing the NPs in ultrapure water and measuring using a Malvern Zetasizer Nano-ZS90 (Malvern Instruments, United Kingdom) at 25 °C. For morphology observation, NPs were diluted to an appropriate concentration, dropped onto a 300-mesh copper grid, stained with 2% (w/v) phosphotungstic acid, and air-dried. Morphology was observed via a transmission electron microscope (TEM, Hitachi, Japan).

### 2.3 Encapsulation efficiency (EE) and loading capacity (LC)

A standard curve of γ-oryzanol was prepared by dissolving γ-oryzanol in anhydrous ethanol to form a 1 mg/mL stock solution, which was then serially diluted to 500, 250, 125, 62.5, 31.25, and 15.63 μg/mL. Absorbance was measured at 327 nm using a Multiskan FC microplate reader (Thermo Fisher Scientific, Shanghai, China), and a standard curve was constructed. For sample measurement, γ-oryzanol@PLGA NP was dissolved in anhydrous ethanol by sonication, centrifuged at 15,000 rpm for 5 min, and the supernatant was analyzed at 327 nm using the same microplate reader. EE and LC were calculated as:

$$\text{EE }\left(\%\right)=\left(W\gamma /W\right)\times 100$$

$$\text{LC }\left(\%\right)=\left(W\gamma /W0\right)\times 100$$

where Wγ is the measured mass of γ-oryzanol in NPs, W is the initial mass of γ-oryzanol, and W0 is the total mass of γ-oryzanol@PLGA NP.

### 2.4 Cellular uptake assay

4T1 breast cancer cells (obtained from the Cell Bank of the Chinese Academy of Sciences, Shanghai, China) were seeded in 6-well plates at 2 × 10^5^ cells/mL (2 mL/well) and cultured in RPMI 1640 medium (Gibco, Grand Island, NY, United States) containing 10% fetal bovine serum (FBS, Gibco, Grand Island, NY, United States) at 37 °C in a 5% CO_2_ incubator for 24 h. The medium was replaced with fresh medium containing C6@PLGA NP or free C6 (equivalent C6 concentration). After incubation for 1 and 4 H, cells were harvested, washed 3 times with PBS, resuspended in 300 μL pre-cooled PBS, and analyzed via a Cytek^®^ Aurora full-spectrum flow cytometer (Cytek Biosciences, United States) (n = 3).

### 2.5 Cytotoxicity assay (CCK-8)

4T1 cells were seeded in 96-well plates at 5 × 10^3^ cells/mL (100 μL/well) and cultured for 24 H. The medium was replaced with 100 μL RPMI 1640 medium (without FBS) containing PLGA NP, free γ-oryzanol, or γ-oryzanol@PLGA NP at various concentrations. After 48 h, 100 μL of 10% Cell Counting Kit-8 (CCK-8, cat. no. C0037, Beyotime Biotechnology Co., Ltd., Shanghai, China) solution was added, and absorbance was measured at 450 nm after 2 h using the Multiskan FC microplate reader. Cell viability was calculated as:

$$\text{Cell viability}\left(\%\right)=\text{Absorbance }\text{of }\text{experimental}\text{group }/\text{Absorbance }\text{of }\text{control }\text{group}\times 100$$

### 2.6 Transcriptomic analysis

4T1 cells were seeded in 6-well plates at a density of 5 × 10^5^ cells/well and cultured in RPMI 1640 medium (Gibco, Grand Island, NY, United States) supplemented with 10% fetal bovine serum (FBS, Gibco) at 37 °C in a 5% CO_2_ incubator for 24 h. After adherence, the medium was replaced with fresh RPMI 1640 medium (without FBS) containing different treatments: (1) γ-oryzanol@PLGA NP (equivalent γ-oryzanol concentration: 50 μg/mL); (2) free γ-oryzanol (50 μg/mL); (3) PLGA NP (50 μg/mL, corresponding to the carrier concentration in the γ-oryzanol@PLGA NP group); (4) PBS (control group). Each group was set with three biological replicates, and cells were incubated for 48 h under the same conditions.

Total RNA was extracted using TRIzol reagent (Invitrogen, Carlsbad, CA, United States) following the manufacturer’s protocol: briefly, cells were lysed with 1 mL TRIzol per well, incubated at room temperature for 5 min, and mixed with 200 μL chloroform. After centrifugation at 12,000×g for 15 min at 4 °C, the upper aqueous phase was transferred to a new tube, mixed with an equal volume of isopropanol, and incubated at −20 °C for 30 min to precipitate RNA. The RNA pellet was washed twice with 75% ethanol (DEPC-treated water), air-dried, and resuspended in 30 μL RNase-free water. RNA concentration and purity were measured using a NanoDrop 2000 spectrophotometer (Thermo Fisher Scientific, Waltham, MA, United States), with A260/A280 ratios required to be between 1.8 and 2.0. RNA integrity was assessed using an Agilent 2,100 Bioanalyzer (Agilent Technologies, Santa Clara, CA, United States), and only samples with an RNA Integrity Number (RIN) ≥ 7.0 were used for subsequent sequencing.

RNA sequencing was performed by Personal Biotechnology Co., Ltd. (Shanghai, China) using an Illumina NovaSeq 6000 platform. Library preparation steps were as follows: (1) mRNA enrichment: poly(A)^+^ RNA was isolated from total RNA using oligo (dT) magnetic beads; (2) fragmentation: mRNA was fragmented into 200–300 bp fragments using divalent cations under elevated temperature; (3) cDNA synthesis: first-strand cDNA was synthesized using random hexamer primers and M-MuLV reverse transcriptase, followed by second-strand cDNA synthesis with DNA polymerase I and RNase H; (4) end repair and adapter ligation: cDNA fragments were subjected to end repair (addition of 3′ A-overhangs) and ligated with Illumina sequencing adapters; (5) PCR amplification: libraries were amplified by PCR with adapter-specific primers to generate final libraries with an average size of ∼350 bp.

Sequencing was conducted in paired-end 150 bp mode, with a sequencing depth of ≥6 Gb per sample. Raw sequencing data (raw reads) were filtered using Trimmomatic (v0.39) to remove low-quality reads (Phred score <20), adapter sequences, and reads shorter than 50 bp, yielding clean reads. Clean reads were aligned to the mouse reference genome (GRCm39) using HISAT2 (v2.2.1) with default parameters, and gene expression levels were quantified as fragments per kilobase of transcript per million mapped reads (FPKM) using featureCounts (v2.0.3).

Differential gene expression analysis was performed using DESeq2 (v1.34.0) in R software. Genes with |log_2_ (fold change)| > 1 and adjusted P-value (padj) < 0.05 (Benjamini–Hochberg method for multiple test correction) were considered significantly differentially expressed. Pathway enrichment analysis of differentially expressed genes was conducted using clusterProfiler (v4.2.2) in R, focusing on Kyoto Encyclopedia of Genes and Genomes (KEGG) pathways. Pathways with a *p*-value <0.05 were considered significantly enriched.

### 2.7 Statistical analysis

Data are presented as mean ± standard deviation (SD). Statistical significance was determined using one-way ANOVA with Tukey’s *post hoc* test. *p* < 0.05 was considered statistically significant.

## 3 Results

### 3.1 Physicochemical characteristics of PLGA nanoparticles

**FIGURE 1:**
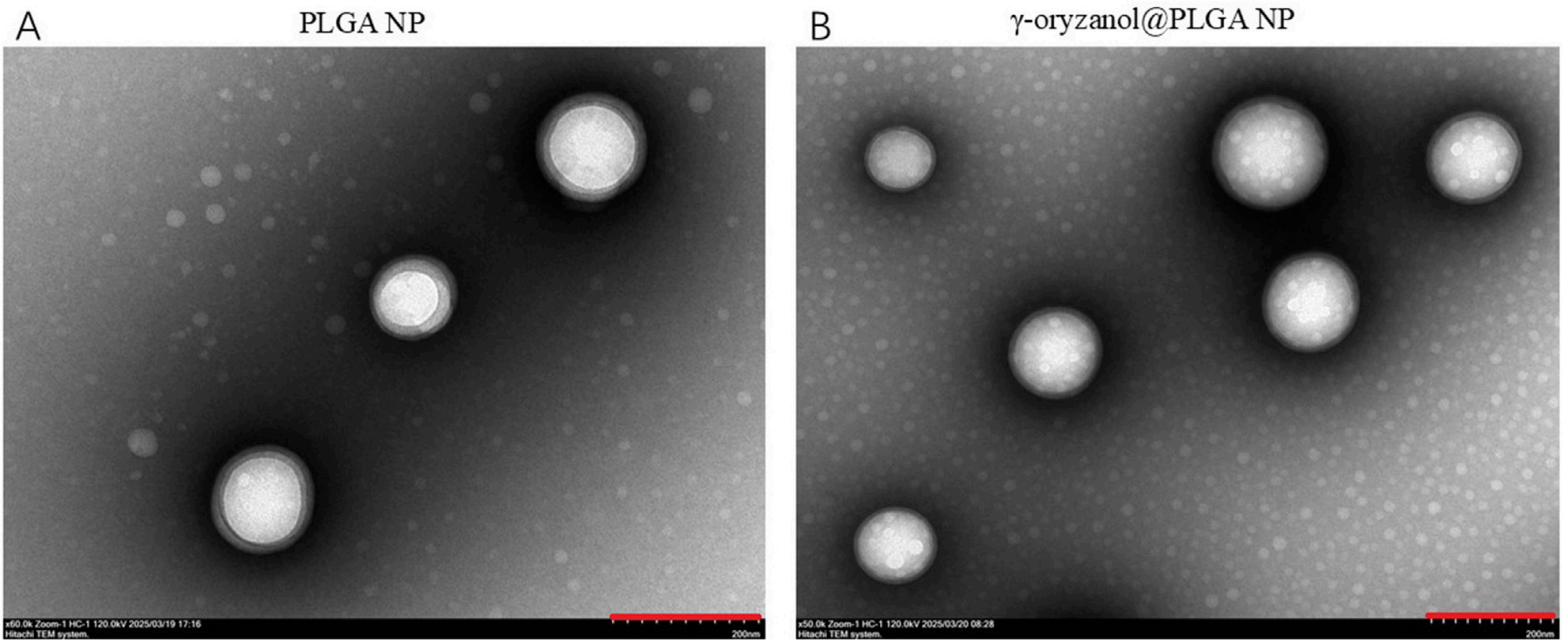
TEM images of PLGA NP **(A)** and γ-oryzanol@PLGA NP **(B)**, Scale bar = 200 nm.

**FIGURE 2:**
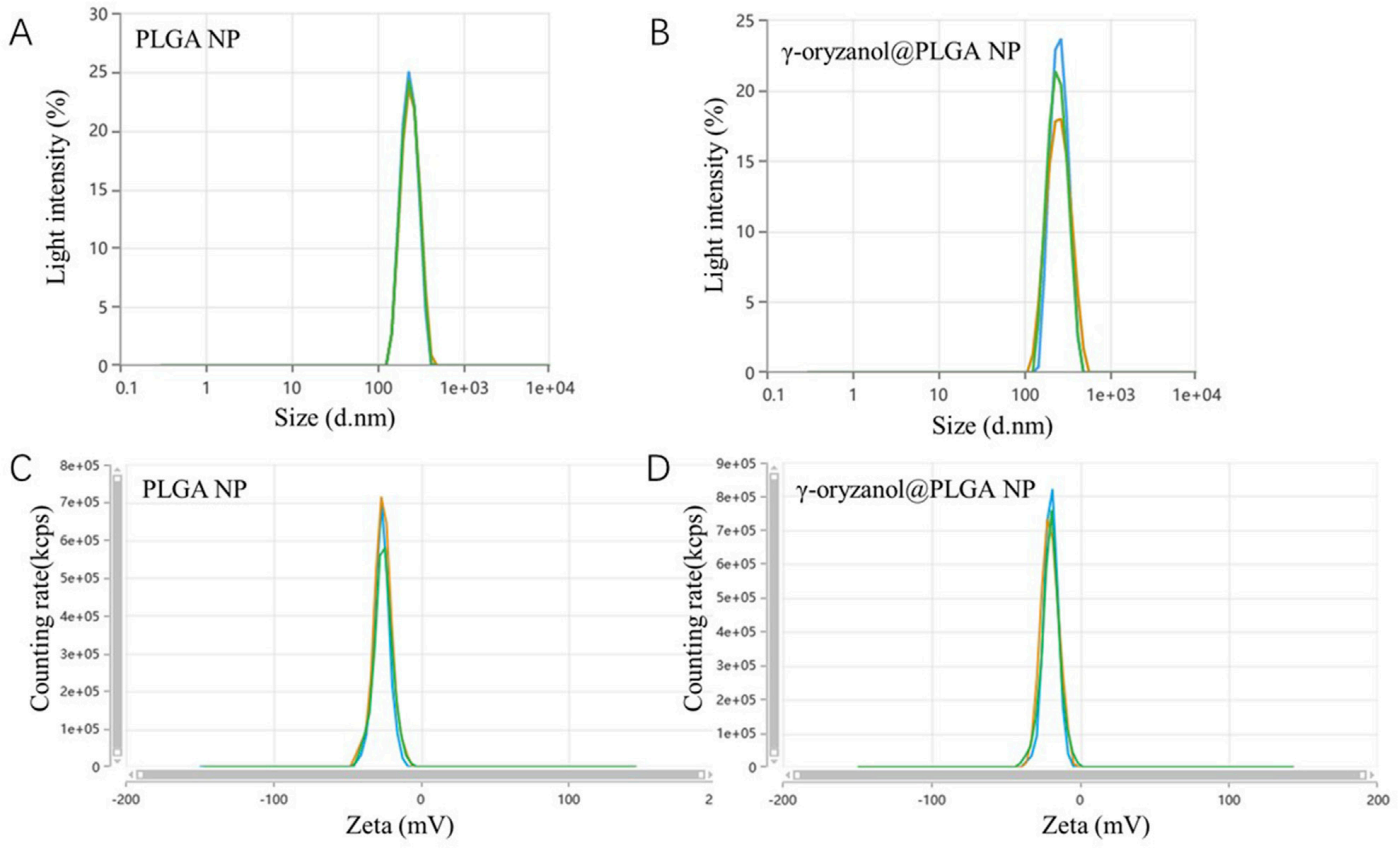
DLS size distribution **(A,B)** and zeta potential **(C,D)** of PLGA NP and γ-oryzanol@PLGA NP.

Transmission electron microscopy (TEM, Figure 1) revealed that both PLGA NP and γ-oryzanol@PLGA NP exhibited spherical morphology with uniform distribution. Dynamic light scattering (DLS) analysis (Figures 2A,B) showed the average hydrodynamic diameters of PLGA NP and γ-oryzanol@PLGA NP were 232.50 ± 1.27 nm and 241.60 ± 7.89 nm, respectively, with polydispersity index (PDI) of 0.016 ± 0.0096 and 0.043 ± 0.035, indicating narrow size distribution. Zeta potential measurements (Figures 2C,D) yielded values of −25.97 ± 0.51 mV (PLGA NP) and −19.84 ± 0.29 mV (γ-oryzanol@PLGA NP), suggesting good colloidal stability.

### 3.2 Encapsulation efficiency and cellular uptake

**FIGURE 3:**
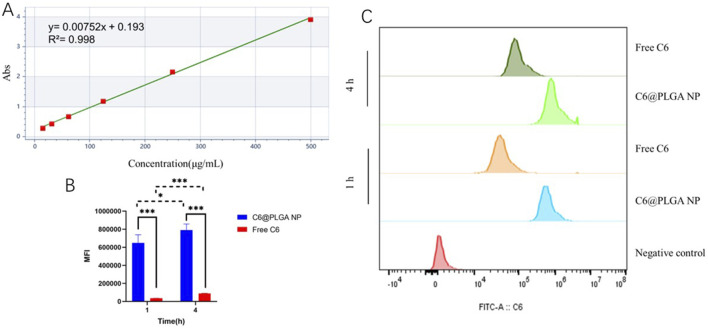
Standard curve of γ-oryzanol **(A)**, mean fluorescence intensity (MFI) of cellular C6 uptake **(B)**, and flow cytometry histograms **(C)**. Data in **(B)** are presented as mean ± SD (n = 3).

The standard curve of γ-oryzanol in anhydrous ethanol showed excellent linearity (y = 0.00752x + 0.193, *R* ^2^ = 0.998, Figure 3A). Based on this curve, the encapsulation efficiency (EE) of γ-oryzanol@PLGA NP was 86.22% ± 3.43%, and the loading capacity (LC) was 7.89% ± 0.31%. For cellular uptake, flow cytometry analysis (Figures 3B,C) demonstrated that the mean fluorescence intensity (MFI) of coumarin 6 (C6) in 4T1 cells treated with C6@PLGA NP was significantly higher than that in cells treated with free C6 at both 1 h and 4 h incubation (*p* < 0.05), indicating enhanced cellular internalization of the nanoparticle-formulated drug (Table 1).

**TABLE 1:** Encapsulation efficiency and drug loading of PLGA nanoparticles.

Group	Size (nm)	PDI	Zeta (mV)	EE%	LC%
PLGA NP	232.50 ± 1.27	0.016 ± 0.0096	−25.97 ± 0.51	-	-
γ-oryzanol@PLGA NP	241.60 ± 7.89	0.043 ± 0.035	−19.84 ± 0.29	86.22 ± 3.43	7.89 ± 0.31

### 3.3 Cytotoxicity of PLGA nanoparticles

**FIGURE 4:**
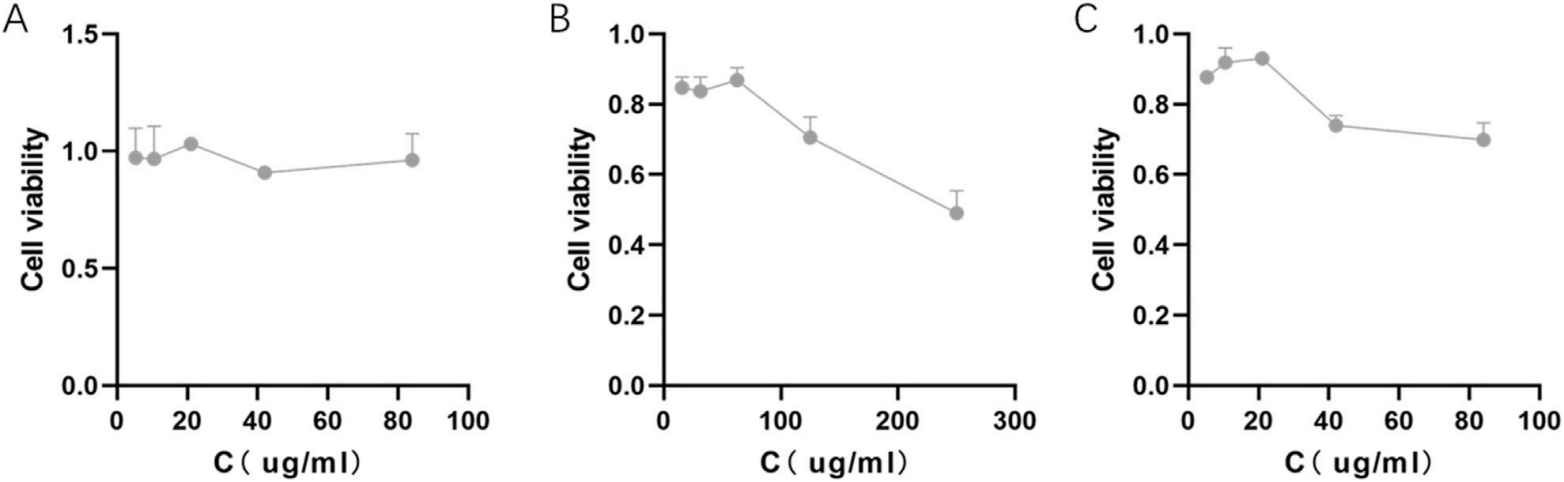
Cytotoxicity of PLGA NP **(A)**, free γ-oryzanol **(B)**, and γ-oryzanol@PLGA NP **(C)** against 4T1 cells. Cell viability was measured by CCK-8 assay. Data are presented as mean ± SD (n = 3).

Cell viability assays (CCK-8) showed that PLGA NP alone had no obvious cytotoxicity against 4T1 cells, with viability remaining >90% across all tested concentrations (Figure 4A). In contrast, γ-oryzanol@PLGA NP exhibited stronger cytotoxicity compared to free γ-oryzanol: the concentration required to reduce cell viability was 42 μg/mL (Figure 4C) for γ-oryzanol@PLGA NP, whereas free γ-oryzanol required 125 μg/mL (Figure 4B), indicating improved bioavailability via PLGA encapsulation.

### 3.4 Transcriptomic multivariate analysis

**FIGURE 5:**
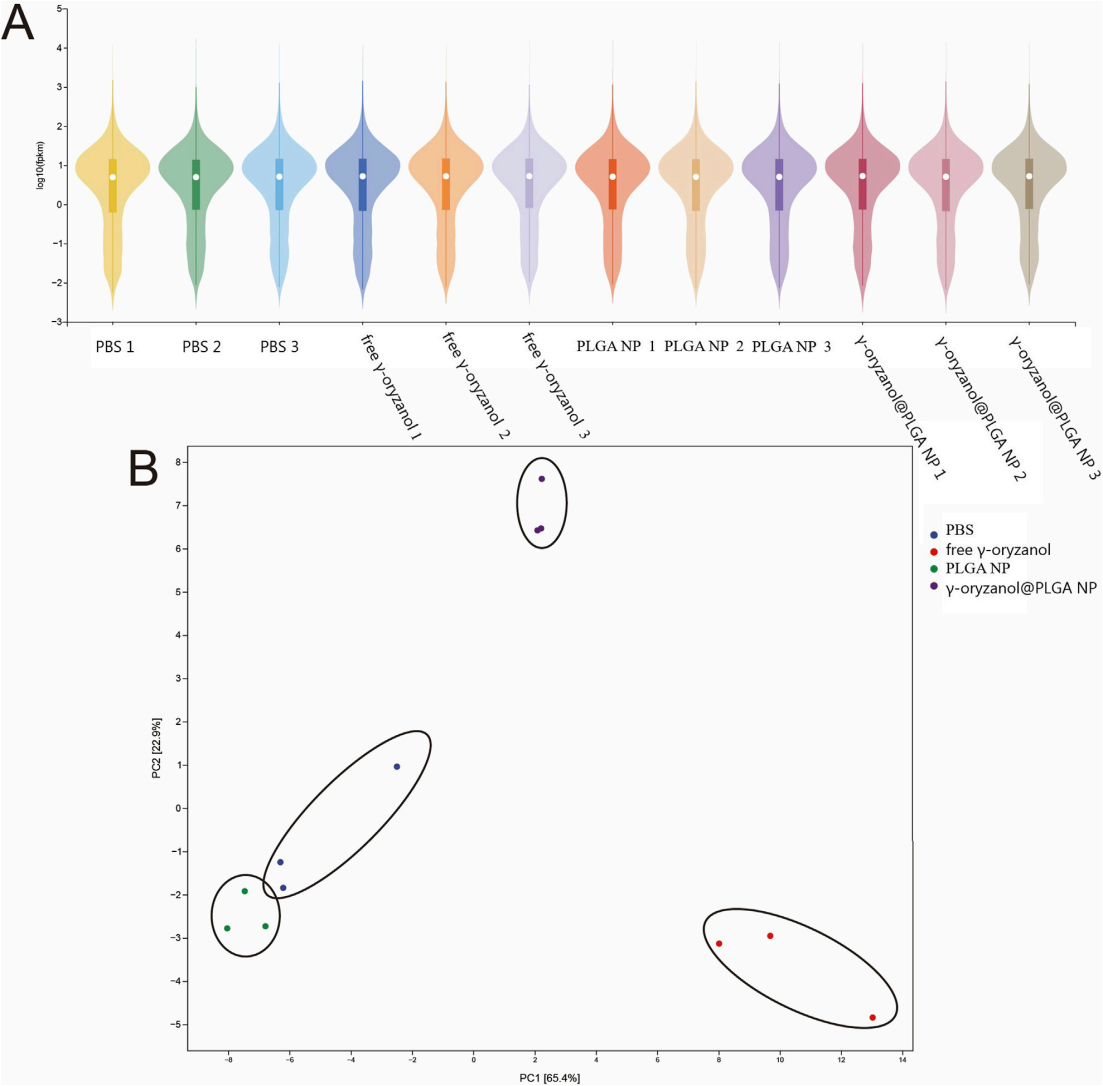
Violin plot **(A)** and PCA **(B)** of transcriptomic profiles across groups.

Violin plots (Figure 5A) illustrated the distribution of molecular features across groups, with the control and PLGA NP groups showing highly similar profiles, confirming the biocompatibility of PLGA. Principal component analysis (PCA, Figure 5B) revealed distinct clustering: the PBS and PLGA NP groups clustered closely, while the free γ-oryzanol and γ-oryzanol@PLGA NP groups formed separate clusters, indicating differential molecular responses induced by different formulations.

### 3.5 Differential gene expression and pathway enrichment across treatment groups

#### 3.5.1 Free γ-oryzanol vs. PBS

**FIGURE 6:**
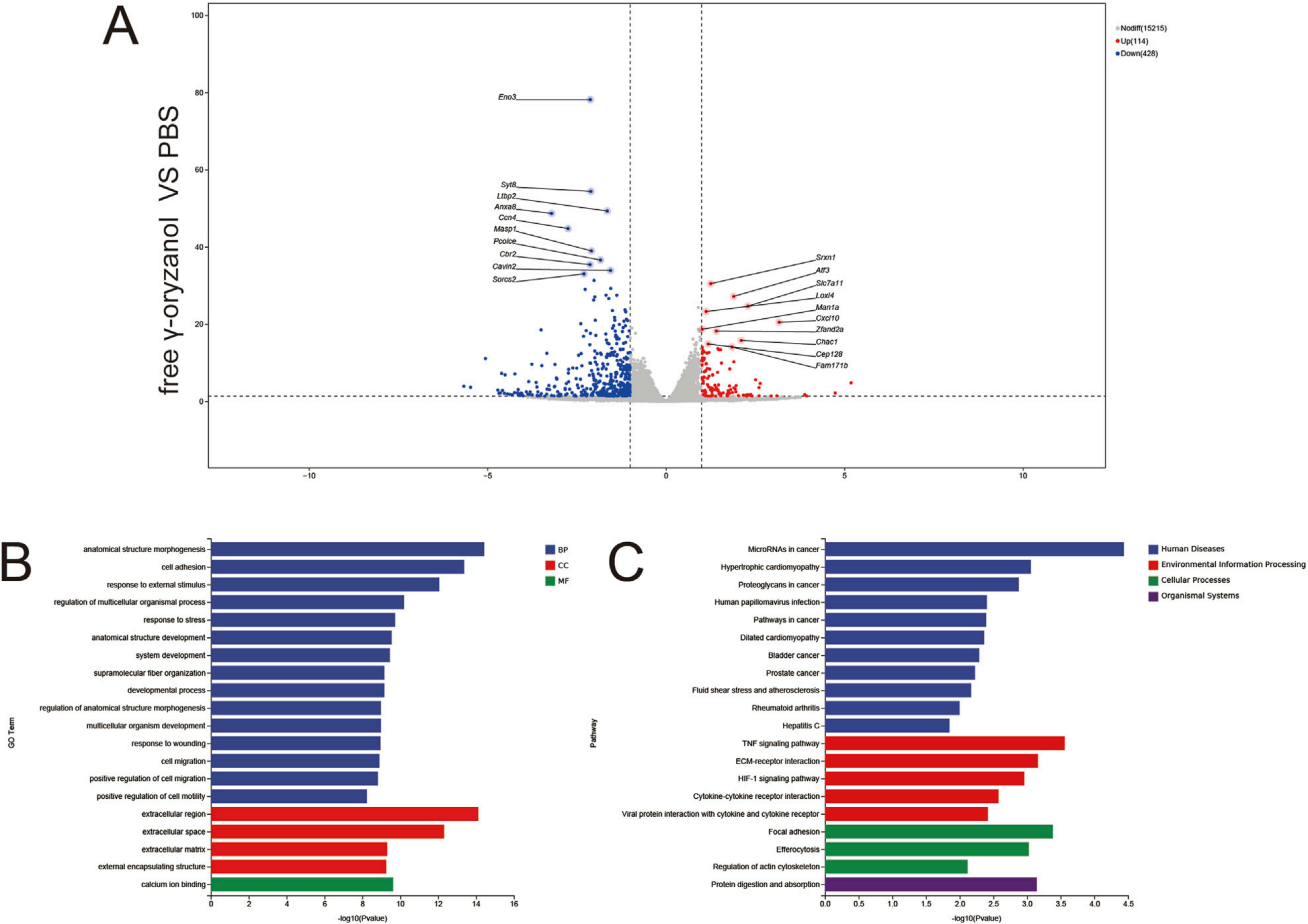
Volcano plots **(A)**, GO enrichment **(B)**, and KEGG pathway analysis **(C)** from independent RNA-seq experiments (free γ-oryzanol VS PBS).

RNA sequencing revealed 1,371 downregulated and 141 upregulated genes (|log_2_FC| > 1, *p* < 0.05, Figure 6A), with key differentially expressed genes (DEGs) including Enox1 (downregulated, oxidative metabolism) and Gpr84 (upregulated, immune signaling). GO Enrichment (Figure 6B) biological Processes (BP): Dominant enrichment in anatomical structure morphogenesis and cell adhesion, reflecting broad cellular structural remodeling. Cellular Components (CC): Focus on the extracellular region, indicating disruption of the extracellular microenvironment. Molecular Functions (MF): Significant enrichment in calcium ion binding, implicating ion homeostasis dysregulation. KEGG Enrichment (Figure 6C) pathways included cytokine–cytokine receptor interaction (immune signaling), focal adhesion (cytoskeletal regulation), and drug metabolism (xenobiotic detoxification), highlighting acute, diffuse transcriptional perturbation by free drug.

#### 3.5.2 PLGA NP vs. PBS

**FIGURE 7:**
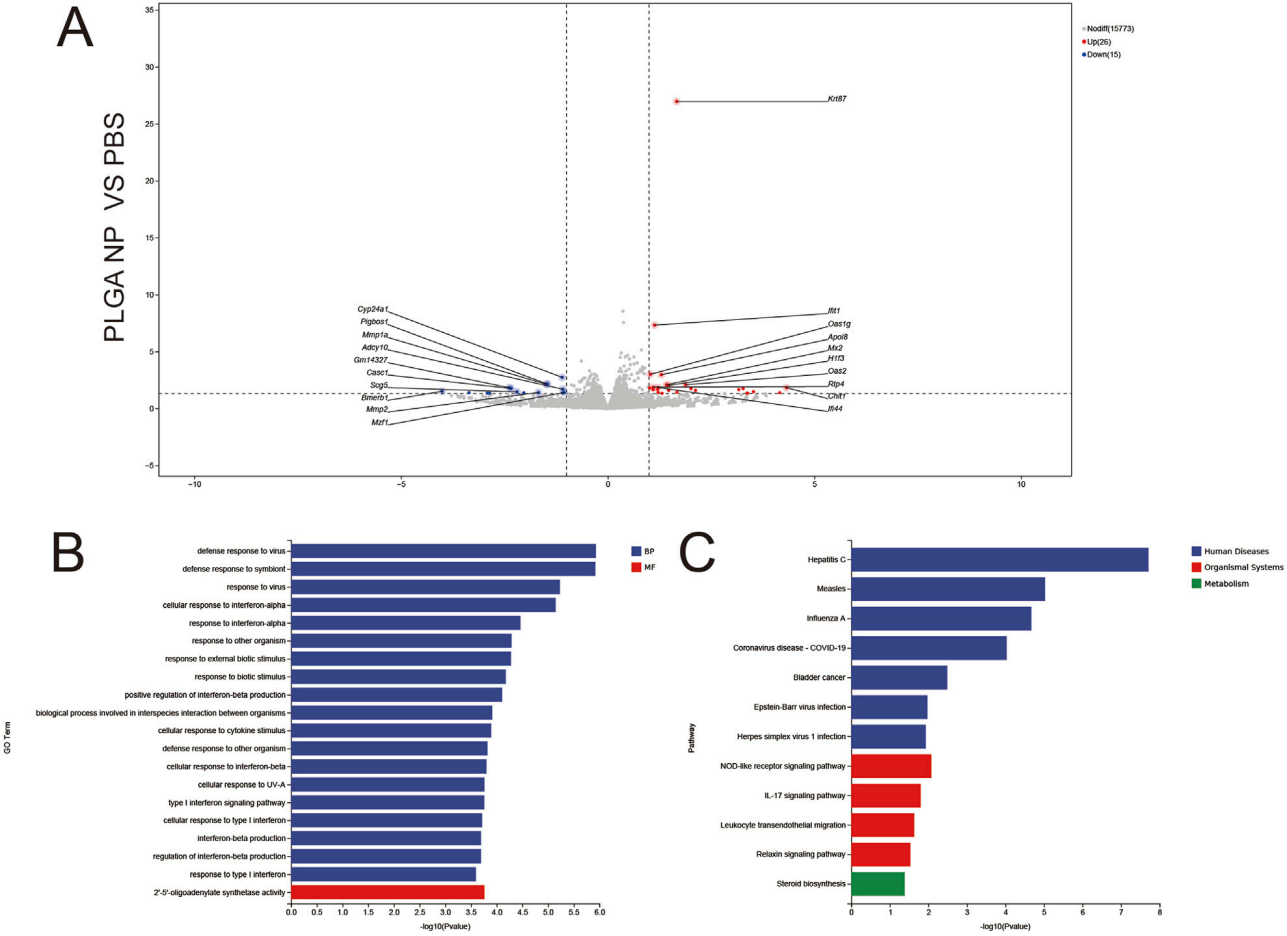
Volcano plots **(A)**, GO enrichment **(B)**, and KEGG pathway analysis **(C)** from independent RNA-seq experiments (PLGA NP VS PBS).

Only 15 downregulated and 26 upregulated genes were detected (Figure 7A), confirming the biocompatibility of PLGA nanoparticles (minimal “foreign body” response). Notable DEGs included Ifit1 and Oasl2 (weak antiviral signaling). GO Enrichment (Figure 7B) BP: Narrow enrichment in defense response to virus and interferon-mediated signaling, reflecting mild innate immune surveillance. MF: Sole enrichment in 2′–5′-oligoadenylate synthase activity, linked to RNA degradation in antiviral responses. KEGG Enrichment (Figure 7C) sparse enrichment in hepatitis C and measles pathways (viral signaling), consistent with minimal transcriptional perturbation by blank PLGA carriers.

#### 3.5.3 γ-oryzanol@PLGA NP vs. PBS

**FIGURE 8:**
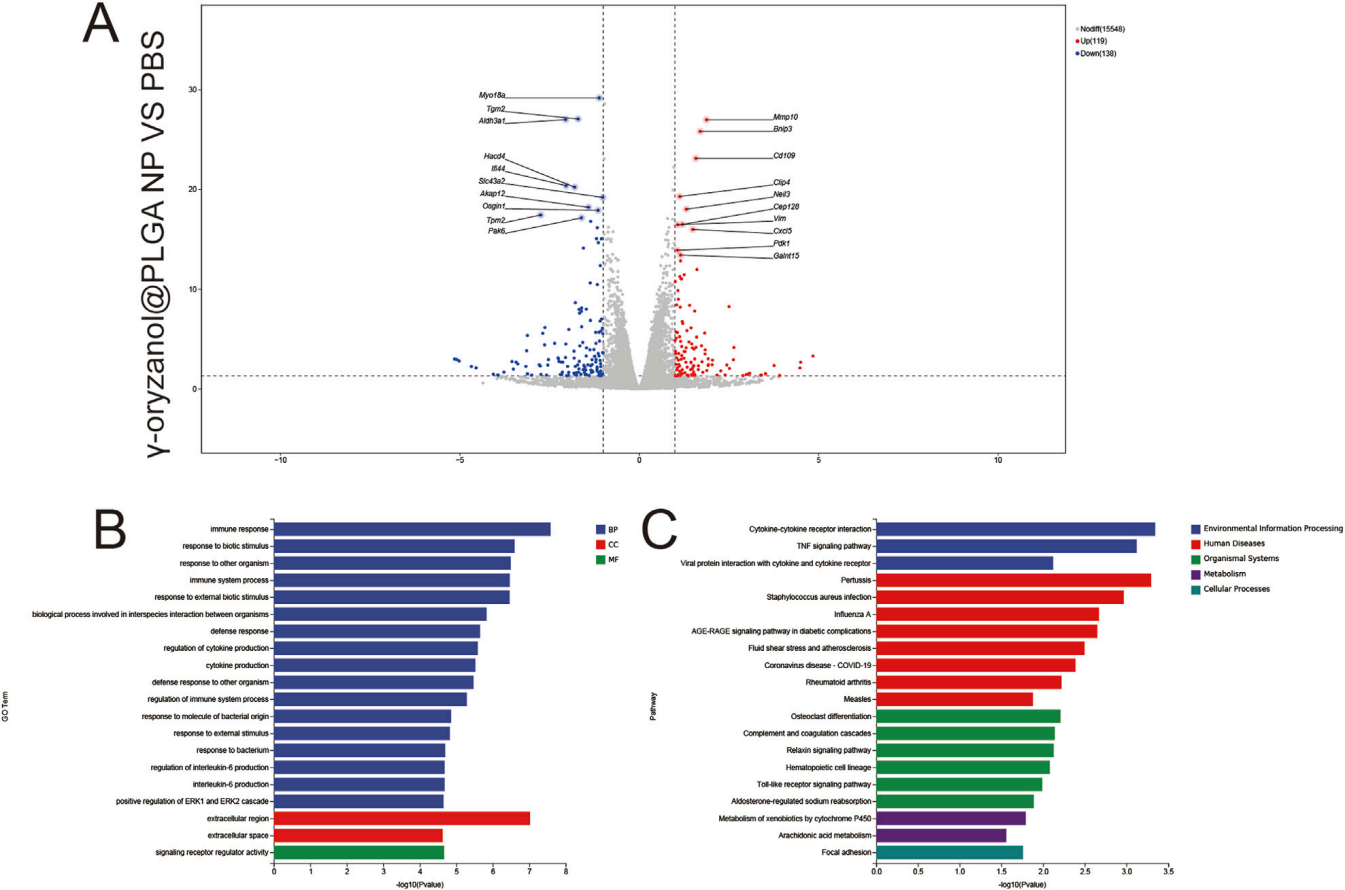
Volcano plots **(A)**, GO enrichment **(B)**, and KEGG pathway analysis **(C)** from independent RNA-seq experiments (γ-oryzanol@PLGA NP VS PBS).

1,811 upregulated and 135 downregulated genes were identified (Figure 8A), with key DEGs including Mmp19 (extracellular matrix remodeling) and Cxcl5 (immune recruitment). GO Enrichment (Figure 8B) BP: Dominant enrichment in immune response and inflammatory response, reflecting targeted immune activation via nanocarrier-mediated endocytosis. CC: Focus on extracellular space and receptor complexes, aligning with focal adhesion–mediated endocytosis of PLGA nanoparticles.

MF: Significant enrichment in signal receptor regulatory activity, indicating receptor-driven signaling cascades. KEGG Enrichment (Figure 8C) pathways included cytokine–cytokine receptor interaction (immune signaling), focal adhesion (endocytic mechanism), and phagosome (efferocytosis), directly linking nanocarrier delivery to immunomodulatory and structural remodeling pathways.

#### 3.5.4 γ-oryzanol@PLGA NP vs. free γ-oryzanol

**FIGURE 9:**
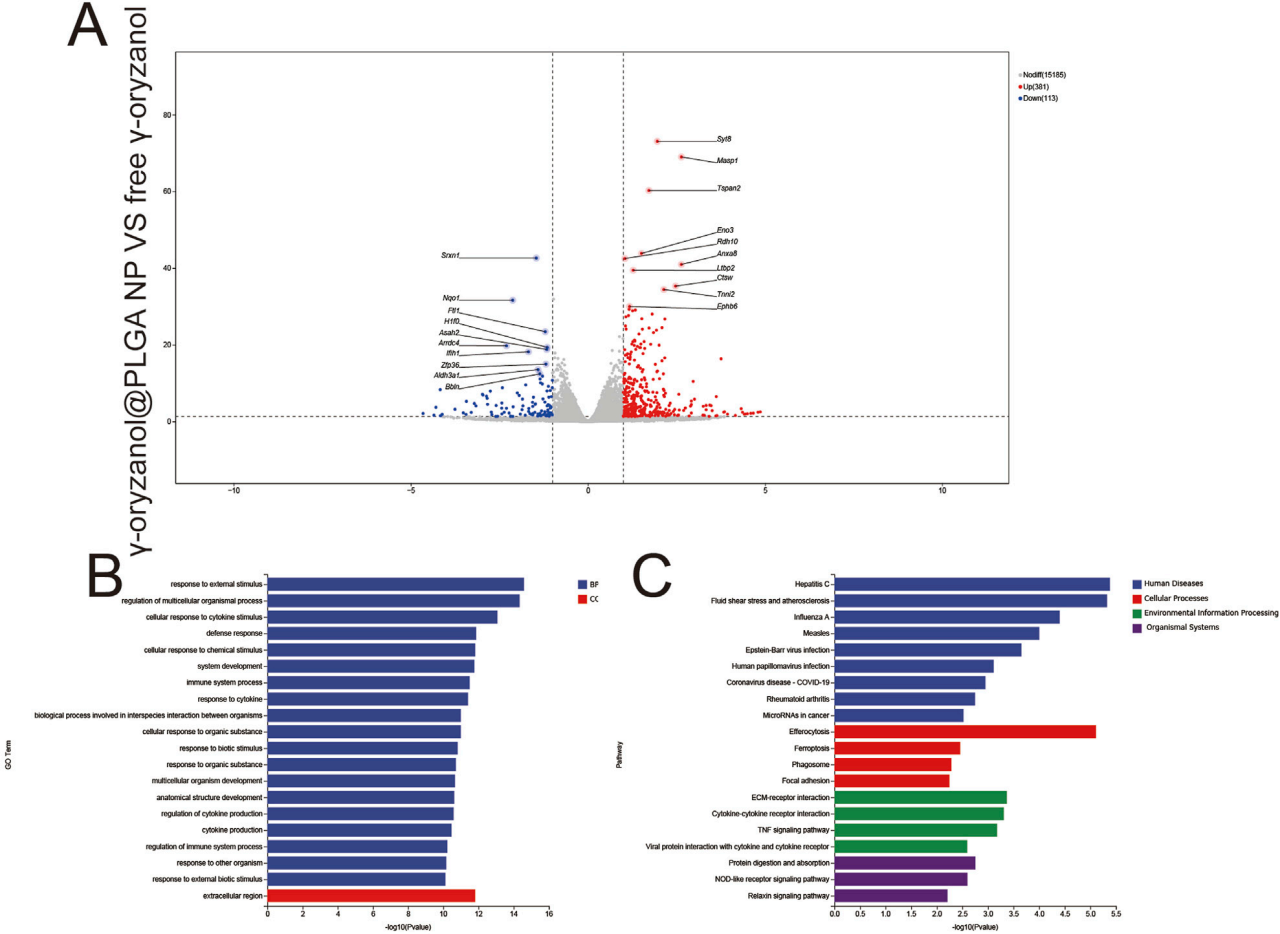
Volcano plots **(A)**, GO enrichment **(B)**, and KEGG pathway analysis **(C)** from independent RNA-seq experiments (γ-oryzanol@PLGA NP VS free γ-oryzanol).

1,316 upregulated and 113 downregulated genes were detected (Figure 9A), revealing fundamental differences in transcriptional regulation between nanocarrier-mediated and free drug delivery. Key DEGs included Smar1 (epigenetic regulation) and Map3k7 (signal transduction). GO Enrichment (Figure 9B) BP: Enrichment in response to chemical stimulus and metabolic process regulation, reflecting sustained metabolic modulation by controlled drug release from PLGA nanoparticles. CC: Focus on the extracellular region, indicating prolonged extracellular matrix remodeling. KEGG Enrichment (Figure 9C) pathways included focal adhesion (enhanced endocytosis), efferocytosis (amplified immunogenic cell death), and the TNF signaling pathway (pro-inflammatory activation), demonstrating the nanocarrier’s ability to amplify immune and structural regulation relative to free drug.

### 3.6 Molecular feature abundance and gene set enrichment analysis (GSEA)

#### 3.6.1 Differential gene expression

Six pairwise comparisons were performed to identify differentially expressed genes (DEGs; |log_2_FC| > 1, FDR <0.05) in Figure 4A PBS vs. free γ-oryzanol: 114 upregulated, 428 downregulated genes. PBS vs. PLGA NP: 26 upregulated, 15 downregulated genes. PBS vs. γ-oryzanol@PLGA NP: 119 upregulated, 138 downregulated genes. free γ-oryzanol vs. PLGA NP: 518 upregulated, 169 downregulated genes. free γ-oryzanol vs. γ-oryzanol@PLGA NP: 381 upregulated, 113 downregulated genes. PLGA NP vs. γ-oryzanol@PLGA NP: 184 upregulated, 224 downregulated genes.

#### 3.6.2 Gene set enrichment analysis (GSEA)

**FIGURE 10:**
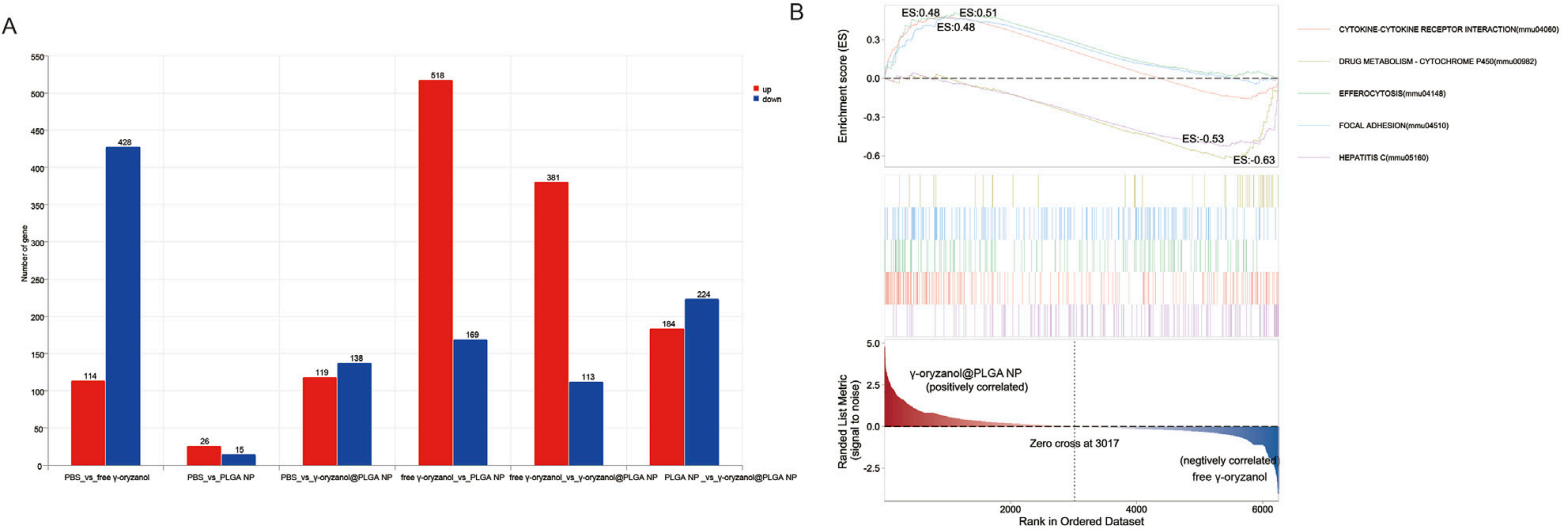
Bar chart depicting the number of differentially expressed genes **(A)** and GSEA results **(B)**.

GSEA revealed pathway-specific enrichment patterns in Figure 4B Positively correlated with γ-oryzanol@PLGA NP: Focal Adhesion (mmu04510, ES = 0.48), Efferocytosis (mmu04148, ES = 0.51), Cytokine-Cytokine Receptor Interaction (mmu04060, ES = 0.48). Negatively correlated with free γ-oryzanol: Drug Metabolism-Cytochrome P450 (mmu00982, ES = −0.63), Hepatitis C (mmu05160, ES = −0.53). The ranked list metric showed a clear separation, with γ-oryzanol@PLGA NP-associated genes enriched in the top-ranked subset and free γ-oryzanol-associated genes in the bottom-ranked subset (Figure 10).

## 4 Discussion

Breast cancer remains a formidable global health challenge, accounting for approximately 30% of all cancer diagnoses in women and representing the leading cause of cancer-related mortality worldwide (Rebecca L et al., 2023). Despite advances in conventional therapies including surgery, chemotherapy, and targeted treatments, significant limitations persist regarding drug resistance, systemic toxicity, and poor bioavailability of therapeutic agents (Ying et al., 2024). The economic burden is substantial, with annual treatment costs exceeding $20 billion in the US alone (Emily D et al., 2023), underscoring the urgent need for innovative therapeutic strategies that can improve treatment efficacy while minimizing adverse effects (Beilei et al., 2024).

This study investigates γ-oryzanol@PLGA nanoparticles as a novel nanotherapeutic approach for breast cancer, building upon emerging evidence of γ-oryzanol’s anticancer properties and PLGA’s established biocompatibility (Soraia et al., 2024). Our systematic evaluation encompasses nanoparticle characterization, cellular uptake dynamics, cytotoxic effects, and transcriptomic profiling to comprehensively assess the therapeutic potential of this nanoformulation. The following discussion interprets key findings regarding nanoparticle physicochemical properties, enhanced drug delivery efficiency, and molecular mechanisms underlying the observed anticancer effects, while contextualizing these results within current nanomedicine paradigms (Yue et al., 2019).

The successful development of γ-oryzanol@PLGA nanoparticles with optimal physicochemical properties represents a significant advancement in nanomedicine formulation. The spherical morphology and narrow size distribution (PDI<0.05) observed through TEM imaging, coupled with negative zeta potentials (−19.84 to −25.97 mV), suggest excellent colloidal stability suitable for systemic administration. Particularly noteworthy is the high encapsulation efficiency (86.22%) achieved through hydrophobic interactions and hydrogen bonding, which compares favorably with reported values for similar natural compound-loaded nanoparticles (Shengjun et al., 2023). The slight increase in particle size after drug loading (232.50–241.60 nm) falls within the optimal range for enhanced permeability and retention effect while maintaining sufficient circulation time. These characteristics address critical formulation challenges in delivering hydrophobic bioactive compounds like γ-oryzanol, potentially overcoming the poor aqueous solubility that has limited its clinical translation.

The remarkable 3.2-fold enhancement in cellular uptake demonstrated at 4H by flow cytometry provides compelling evidence for the superiority of nanoparticle-mediated delivery over free drug administration (Yuko and Tatsushi, 2012). This observation aligns with established principles of nanoparticle-cell interactions, where the endocytic uptake mechanisms (likely clathrin-mediated endocytosis given the particle size) bypass efflux pumps and other membrane barriers that typically limit intracellular accumulation of hydrophobic drugs (Sulin et al., 2015). The time-dependent increase in fluorescence intensity suggests progressive internalization rather than mere surface adsorption. These findings have important implications for overcoming multidrug resistance, a major challenge in cancer chemotherapy (Chunyan et al., 2023). The differential uptake kinetics between free drug and nanoparticle formulations may explain the subsequent differences in cytotoxic effects observed in our study, though further investigation of intracellular trafficking and drug release profiles would strengthen these conclusions.

Our cytotoxicity results demonstrate a three-fold reduction in the minimum effective concentration when γ-oryzanol is delivered via PLGA nanoparticles compared to free drug (42 vs. 125 μg/mL), while maintaining excellent biocompatibility (>90% viability for blank nanoparticles). This enhanced potency likely results from multiple factors: improved cellular internalization as shown in uptake studies, controlled intracellular drug release from the biodegradable polymer matrix, and potential protection of γ-oryzanol from enzymatic degradation or efflux (Jing et al., 2024). The concentration-dependent response suggests maintained pharmacological activity of the encapsulated compound (Dinesh C and Anthony, 2021), while the absence of cytotoxicity from blank nanoparticles confirms the safety profile of the delivery system itself (Nastassja et al., 2008). These findings are particularly promising given that γ-oryzanol’s mechanism involves modulation of multiple signaling pathways rather than simple cytotoxic effects, suggesting the nanoparticle formulation preserves the compound’s complex bioactivity while improving its pharmacokinetic properties (Wiramon et al., 2019).

Transcriptomic analysis revealed profound molecular effects of γ-oryzanol@PLGA treatment, with 576 differentially expressed genes including key regulators like Stat1 and Irf1. Pathway enrichment analysis identified significant involvement of cancer-related pathways (*p* = 3.2e-5), TNF signaling (*p* = 1.8e-4), and actin cytoskeleton regulation (*p* = 4.7e-3), providing mechanistic insights into the enhanced therapeutic effects observed (Bharat B, 2003; G et al., 2020; Hon Yan Kelvin and Antonella, 2021). The upregulation of interferon-related genes (Stat1, Irf1) suggests activation of antitumor immune responses (Ling et al., 2023), while downregulation of metabolic enzymes like Enox1 may indicate metabolic reprogramming of cancer cells (Linchong et al., 2021). These multi-target effects are characteristic of natural compounds and may explain the superior efficacy compared to single-target agents (Abbas and Aaron, 2022). The minimal transcriptomic changes induced by blank PLGA nanoparticles further confirm their biological inertness and safety as drug carriers (Anthony et al., 2021). These findings not only validate the therapeutic potential of γ-oryzanol but also provide a molecular signature for future biomarker development and combination therapy strategies.

The methodological rigor demonstrated in this study, including excellent linearity (*R* ^2^ = 0.998) in drug quantification and minimal batch-to-batch variation (<5%), establishes robust protocols for reproducible nanoparticle production. The standardized preparation method using PVA as stabilizer yields particles with consistent size distribution and drug loading characteristics suitable for scale-up (J and A, 2002). However, translation to clinical applications would require addressing several considerations: optimization of sterilization methods without compromising nanoparticle stability (Melissa A et al., 2014), comprehensive stability testing under various storage conditions (Rubén et al., 2019), and development of lyophilization protocols for long-term storage (Yuan et al., 2025). The current formulation meets many critical quality attributes for nanomedicines, including particle size control, high drug loading, and colloidal stability, positioning it well for preclinical development. Future work should focus on establishing quality control parameters for Good Manufacturing Practice (GMP) compliance and investigating potential interactions with biological components *in vivo* that might affect performance (Cintia et al., 2023).

While this study demonstrates promising results in developing γ-oryzanol-loaded PLGA nanoparticles, several limitations should be acknowledged. The absence of *in vivo* pharmacokinetic and pharmacodynamic data restricts our understanding of the formulation’s systemic behavior and therapeutic potential in complex biological systems. Furthermore, the single cell line model (4T1 murine breast cancer cells) may not fully recapitulate the heterogeneity of human breast cancers, limiting the generalizability of our findings. The transcriptomic analysis, while revealing important molecular pathways, would benefit from orthogonal validation of key differentially expressed genes (e.g., Stat1, Irf1) through qPCR or Western blot analysis to strengthen the mechanistic insights.

## 5 Conclusion

In conclusion, this work successfully establishes γ-oryzanol@PLGA NPs as a stable, biocompatible nanocarrier system with enhanced cellular uptake and cytotoxic effects against breast cancer cells. The comprehensive physicochemical characterization, coupled with transcriptomic profiling of molecular pathways, provides a solid foundation for further development of this nanoformulation. Future studies should prioritize *in vivo* efficacy evaluation, investigation of immune modulation effects, and exploration of combination therapies to advance this promising therapeutic strategy toward clinical translation.

## Data Availability

The datasets presented in this study can be found in online repositories. The names of the repository/repositories and accession number(s) can be found in the article/Supplementary Material.
